# Disposable Stochastic Sensors Based on Nanolayer Deposition(s) of Silver and AgC Composite on Plastic for the Assay of α-amylase in Whole Blood and Saliva

**DOI:** 10.3390/nano10081528

**Published:** 2020-08-04

**Authors:** Raluca-Ioana Stefan-van Staden, Alexandrina Moscalu-Lungu, Marius Badulescu

**Affiliations:** 1 Laboratory of Electrochemistry and PATLAB Bucharest, National Institute of Research for Electrochemistry and Condensed Matter, 060021 Bucharest, Romania; 2 Faculty of Applied Chemistry and Material Science, Politehnica University of Bucharest, 060021 Bucharest, Romania; alex_ly15@yahoo.com; 3 Low Temperature Plasma Laboratory, National Institute for Lasers, Plasma and Radiation Physics (NILPRP), 409 Atomistilor St., 077125 Magurele, Romania

**Keywords:** α-amylase, disposable stochastic sensors, nanolayer deposition(s), bioanalysis

## Abstract

Nanolayer deposition(s) of Ag and AgC composite on a plastic substrate was used to design disposable stochastic sensors. Three shapes of nanocoatings were tested. The first shape was obtained by deposition of a nanofilm of Ag on the plastic material; the second shape was obtained by deposition of a composite AgC nanolayer on the plastic material; the third shape was obtained by nanolayer deposition of a composite material of AgC on the top of the Ag nanofilm deposited on the plastic material. α-Cyclodextrin was used to modify the active surface of the sensor. Wide linear concentration ranges were obtained as follows: for de-assay of α-amylase in whole blood: 1.00 × 10^−7^–1.00 × 10^3^ U mL^−1^ and for the assay of α-amylase in saliva: 1.5 × 10^−15^–1.5 × 10^2^ U mL^−1^. α-Amylase was reliably determined from whole blood and saliva samples using the proposed disposable stochastic sensors.

## 1. Introduction

α-Amylase has become an enzyme of high importance as a biomarker for diabetes and pancreas-related diseases [1,2,3,4]. Salivary amylase is secreted by the salivary glands and is released primarily through adrenergic innervation, while pancreatic amylase in blood is mainly produced and released by the pancreas [2]. The level of blood amylase concentrations must be low and constant and can increase in acute pancreatitis and salivary gland inflammation [4]. Inhibition of α-amylase activity can decrease the appearance of type II diabetes; it is an important drug target for the treatment of diabetic patients. The reference values for α-amylase are: total serum amylase lower than 100 U L^−1^; pancreatic amylase between 13 and 53 U L^−1^; and salivary amylase lower than 47 U L^−1^.

Different methods such as spectrophotometry, chromatography and immunology have been proposed to determine the α-amylase. However, the disadvantage of these techniques is that they are sensitive to colorimetric turbidity of the samples [5]. Furthermore, electrochemical sensors are simple, accurate, low-cost and sensitive [6,7]. Therefore, α-amylase activity in human serum has been detected with an oligosaccharide dehydrogenase-modified graphite paste electrode [8]. Moreover, another electrochemical method used for the assay of α-amylase activity has been suggested based on the determination of α-amylase-generated maltose using a peroxide electrode equipped with glucose oxidase, α-glucosidase and mutarotase immobilized on cellophane membrane [9].

The aim of this study was to develop three disposable stochastic sensors. Nanolayer deposition(s) of Ag and AgC composite on a plastic substrate was used to design the new disposable stochastic sensors. α-CD was used as a modifier because it is able to provide the required channels needed in stochastic sensing.

Stochastic mode was chosen for these determinations due to its advantages versus classical modes such as differential pulse voltammetry: very small concentrations of biomarkers can be determined in very complex matrices such as biologic samples, because the response of the stochastic sensors is not influenced by the composition of the matrix; also, no pretreatment of the samples was needed [10,11,12]. Selection of nanomaterials for sensing devices proved earlier to improve the quality and reliability of the analytical information obtained using different types of sensors [13,14,15].

## 2. Experimental

### 2.1. Materials and Reagents

All reagents were of analytical grade. The solutions were prepared using deionized water. Pancreatic and salivary α-amylase, α-cyclodextrin (α-CD) were purchased from Sigma-Aldrich (Milwaukee, USA) and paraffin oil from Fluka (Buchs, Switzerland). Phosphate buffer solution (PBS, 0.2 mol L^−1^) with pH = 7.5 was prepared using NaH_2_PO_4_ and Na_2_HPO_4_ (from Sigma-Aldrich). A stock solution of pancreatic (from human pancreas) α-amylase (PAA) with a concentration of 1.00 × 10^3^ U L^−1^, respectively a solution of salivary α-amylase (from human saliva) (SAA) with a concentration of 1.5 × 10^2^ U mL^−1^ buffered with PBS (pH = 7.5) were used to obtain the sets of solutions needed for the calibration of the disposable stochastic sensors, using the serial dilution method.

### 2.2. Apparatus

Direct-Q3 water purification system (Millipore Corporation, Paris, France) was used to prepare deionized water. An AUTOLAB/PGSTAT 302 N (Metrohm, Utrecht, The Netherlands), connected to a computer with GPES software was used for all the electrochemical measurements. A three-electrode system containing Ag/AgCl ink as reference electrode, platinum ink as counter electrode and the disposable stochastic sensor was used for all measurements.

### 2.3. Design of Disposable Stochastic Sensors

**Scheme 1:**
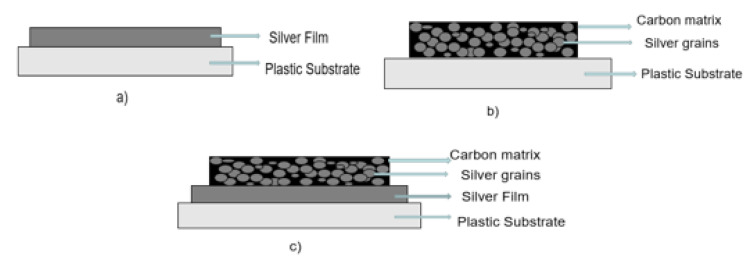
Configuration of the disposable stochastic sensors based on (**a**) silver nanofilm; (**b**) AgC composite nanofilm; and (**c**) AgC composite nanofilm deposited on a Ag nanofilm.

All coatings were made onto plastic substrates (overhead projector transparency film- suitable for use with photocopiers and laser printers). Before deposition, the substrates were ultrasonically cleaned, washed with deionized water and blown by nitrogen in order to remove any contaminants and to increase the surface functionalities. Nanoarchitecture coatings were synthesis based on two materials with high purity (Ag = 99.99%, C = 99.98%) used in design for sensor applications (Scheme 1).

**Scheme 2:**
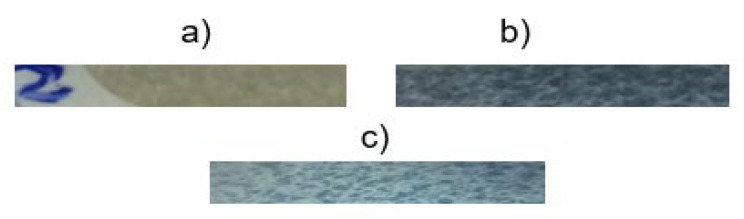
Digital photos of stochastic sensors based on (**a**) Ag nanofilm; (**b**) AgC composite nanofilm; and (**c**) AgC composite nanofilm deposited on a Ag nanofilm.

Three shapes of nanocoatings were tested: the first shape was obtained by deposition of a nanofilm of Ag on the plastic material (Scheme 1a); the second shape was obtained by deposition of a composite AgC nanolayer on the plastic material (Scheme 1b); the third shape was obtained by nanolayer deposition of a composite material of AgC on the top of the Ag nanofilm deposited on the plastic material (Scheme 1c). Digital photos of nanofilms deposited onto plastic substrate are shown in Scheme 2.

All depositions were performed using cold plasma at room temperature, in high vacuum. The plastic substrates were placed at 33-cm distance from anode to achieve the optimum plasma coatings. The total deposition time for each layer was 5 min. Prior to each coating, the chamber was evacuated to a pressure of 4 × 10^−6^ mBar. The anode was designed especially for the deposition of AgC composite nanofilms and consists of Ag and C base. The deposition was done using one– phase layered coatings strategy. Briefly, the designed anode is a graphite crucible (electrically coupled) containing both materials to be evaporated. In the crucible, a carbon rod (ϕ7 × 10 mm) was positioned and fully surround with 5 g of solid silver. Single stable plasma plume from both materials (Ag and C) was ignited and maintained by using electron bombardment from a single hot tungsten filament.

A solution of α-cyclodextrin 10^−3^ mol L^−1^ was used as a modifier in the design of new disposable stochastic sensors based on Ag, AgC and AgC/Ag coatings materials onto plastic substrates. As shown in previous publications [16,17,18], 10^−3^ mol L^−1^ was the optimum concentration of modifier (α-cyclodextrin); using higher or lower concentrations of α-cyclodextrin, no response was obtained for the proposed sensors.

When being used for calibration graphs, it was possible to use the same sensor for the entire linear concentration range; in this case, before each measurement, the new stochastic sensors were cleaned with deionized water. When not in use, they were kept at room temperature in a dry place.

**Figure 1:**
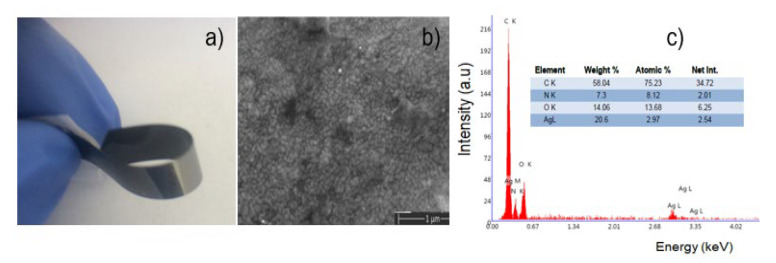
Nanoarchitecture thin films deposited on plastic material. (**a**) digital image of AgC/Ag nanoarchitecture coatings; (**b**) top-view scanning electron microscope (SEM) image of (**a**); (**c**) EDAX spectrum and elemental composition of (**b**).

Characterization of the sensors was done using EDAX, SEM and AFM. The digital image of AgC/Ag nanoarchitecture coating is shown in Figure 1a. An SEM image of the AgC/Ag nanoarchitecture is shown in Figure 1b. EDAX spectra presented in Figure 1c are consistent with the proposed composition. These results of EDAX elemental microanalysis of the AgC/Ag are listed in Figure 1c.

**Figure 2:**
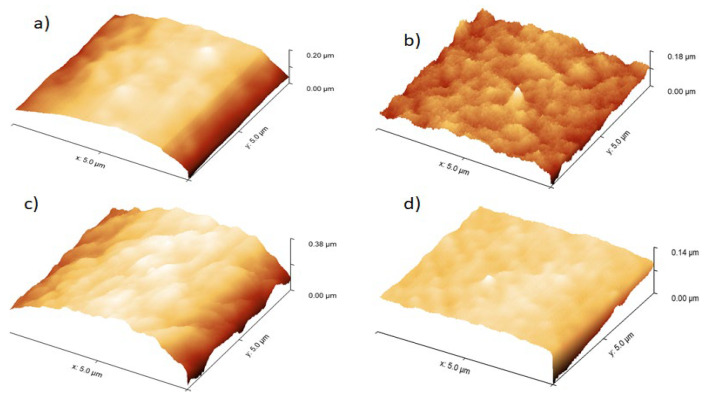
Three-dimensional images obtained using the AFM for the (**a**) plastic substrate, (**b**) Ag nanofilm, (**c**) AgC nanocomposite film and (**d**) AgC/Ag nano-architecture coating.

The average roughness of the substrates was measured using an atomic force microscope (AFM) which provided the following root mean square (RMS) values: (a) 32 nm for plastic substrates, (b) 14-nm for Ag nanofilm, (c) 10 nm for AgC nanocomposite film and (d) 8.2 nm for AgC/Ag nanoarchitecture coatings. One can observe a decrease in surface roughness after coating process the lowest value being registered for the AgC/Ag nanoarchitecture coating. Three-dimensional images of plastic substrate are shown in Figure 2 along the Ag, AgC, AgC/Ag nanocoatings.

### 2.4. Stochastic Mode

For the stochastic mode a chronoamperometric technique was selected, for which a constant potential (125 mV versus Ag/AgCl) was applied. 

**Figure 3:**
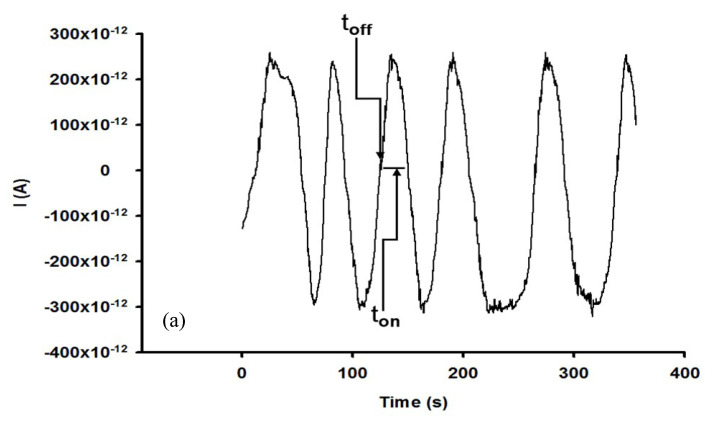

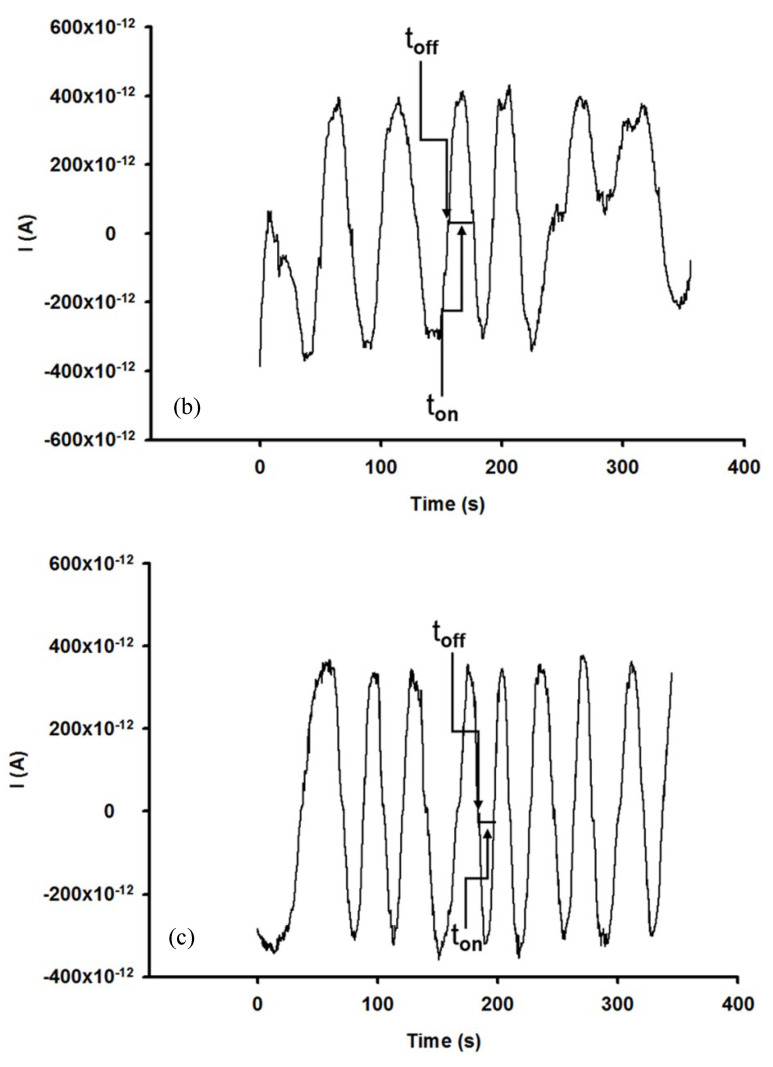
Examples of diagrams obtained when the disposable stochastic sensor based on: (**a**) α-CD/Ag; (**b**) α-CD/AgC; (**c**) α-CD/AgC/Ag used for the measurement of α-amylase in whole blood samples.

**Figure 4:**
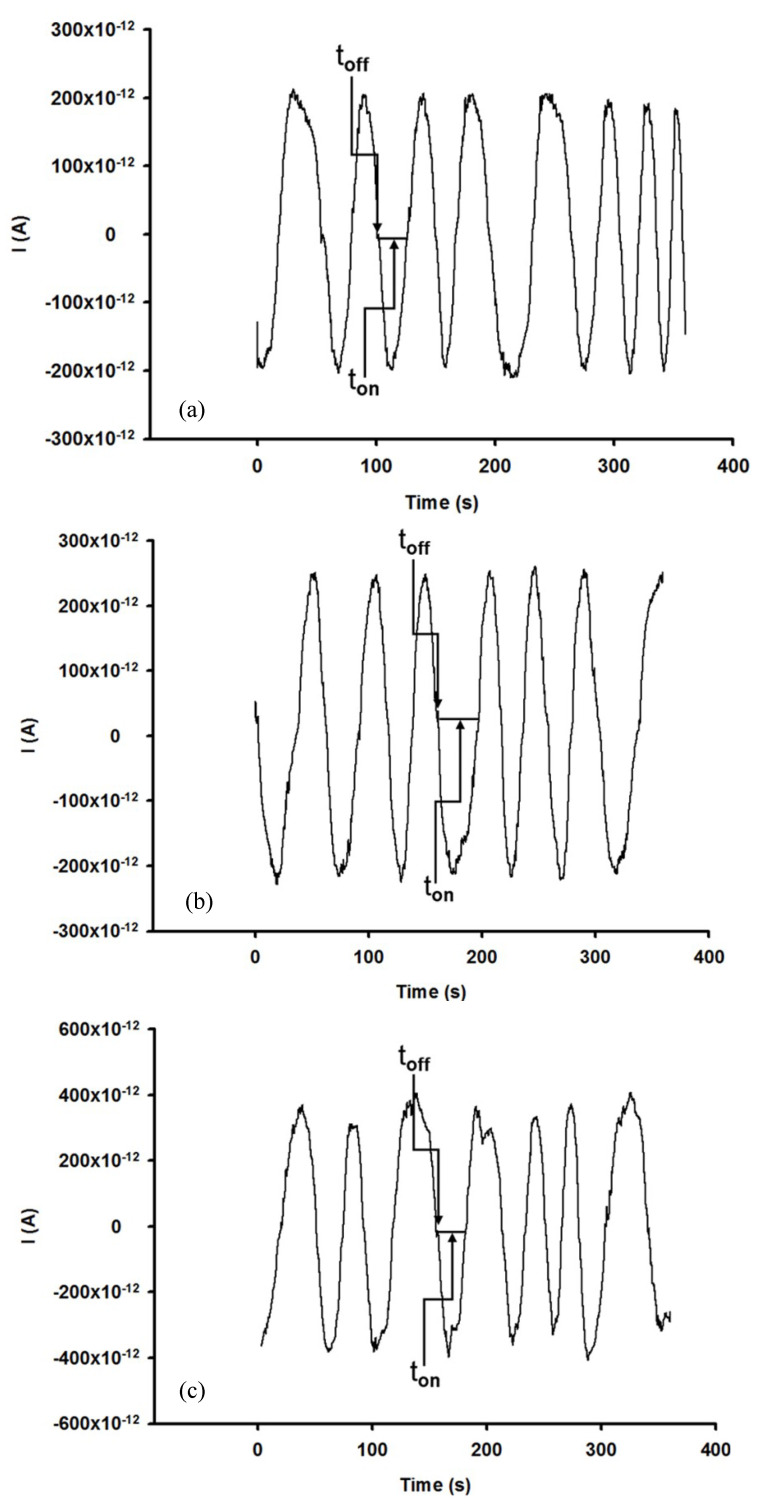
Examples of diagrams obtained when the disposable stochastic sensor based on: (**a**) α-CD/Ag; (**b**) α-CD/AgC; (**c**) α-CD/AgC/Ag used for the measurement of α-amylase in saliva samples.

The calibration was performed using standard solutions with different types of α-amylase: pancreatic α-amylase (PAA) and salivary α-amylase (SAA). The molecular recognition of pancreatic and salivary α-amylase (t_off_ values), and the quantitative assay (t_on_ values) were assigned from the stochastic diagrams (Figure 3 and Figure 4). In order to find unknown amounts of pancreatic and salivary α-amylase from biologic fluids, the values of t_on_ were inserted in the equations of calibration: 1/t_on_ = a + b × Conc._α-amylase_ obtained using the linear regression method. 

### 2.5. Samples 

Ten biologic samples were used for the validation of the method—five whole blood samples and five saliva samples. The whole blood and saliva samples were investigated as collected from the patients. Informed consent was obtained from the patients for the biologic samples, accordingly with the approved Ethics Committee of the University of Medicine and Pharmacy “Carol Davila” from Bucharest, document nr 11/2013.

## 3. Results and Discussions

### 3.1. Response Characteristics of the Stochastic Sensor Used for the Determination of Pancreatic (PAA) and Salivary (SAA) α-Amylase

Three new disposable stochastic sensors based on nanolayer deposition(s) of Ag, AgC and AgC on Ag onto plastic were modified with α-CD and characterized using stochastic method. The response of the stochastic sensors was based on channel conductivity: in the first stage, the molecule of α-amylase is blocking the channel, while the intensity of the current is dropping to zero value; the period of time spent at this stage is represented by the signature (t_off_) of α-amylase this was the qualitative parameter used for the identification of the signal specific to pancreatic/salivary α-amylase in the recorded diagrams (Figure 3 and Figure 4). The second stage of the stochastic mode is related to the quantitative assay of the pancreatic/salivary α-amylase while the molecule is binding with the wall of the channel and the redox processes are taken place, this period of time is known as ton (Figure 3 and Figure 4) and it was used for the quantitative measurements. The response characteristics of the stochastic sensors were recorded for PAA and SAA. These are shown in Table 1 and Table 2. All values shown in Table 1 and Table 2 are the average of ten determinations (10 calibrations were performed for each sensor).

For the PAA, the linear concentration ranges for all three stochastic sensors were between 1 × 10^−7^ and 1 × 10^3^ U L^−1^ and the limit of determination of 1 × 10^−7^ U L^−1^. The highest sensitivity (2.49 × 10^3^ s^−1^/U L^−1^) was recorded using the stochastic sensor based on a single nanofilm of Ag on plastic (Table 1). For SAA the wider linear concentration ranges (10^−15^–10^2^ U mL^−1^) and the lowest limit of determination (10^−15^ U mL^−1^) were recorded when the stochastic sensor based on AgC nanofilm and stochastic sensor based on nanolayer-by-nanolayer deposition of AgC/Ag on plastic were used; the highest sensitivity (7.07 × 10^8^ U mL^−1^) was obtained for the sensor based on the deposition of Ag nanofilm on plastic. The limits of determination were determined accordingly with the definition given by IUPAC– the lowest concentration situated on the linear concentration rage.

### 3.2. Selectivity of the Stochastic Sensors

The selectivity is given by the t_off_ values (signatures) recorded for the other supposed interference species. If the signatures of the supposed interference species are different from those recorded for PAA and SAA, they do not interfere. The following interference species were selected: CEA, CA199, proinsulin, D-glucose. All these species had different signatures than PAA and SAA, proving that the disposable stochastic sensors are selective.

### 3.3. Analytical Applications

The stochastic method and the new stochastic sensors were validated using whole blood and saliva samples. The first step in the validation process was the recovery of PAA and SAA in whole blood and saliva samples, respectively. In this regard, PAA and SAA were determined from whole blood and saliva samples, respectively. To each of the whole blood, and saliva samples were added different amounts of PAA (in the whole blood) and SAA (in the saliva). Recoveries, % of the amounts added are shown in Table 3 and Table 4—higher values than 99.00% were obtained, proving that PAA and SAA can be determined from biologic samples.

Further, five whole blood samples and five saliva samples were analyzed using the proposed disposable stochastic sensors. Table 5 and Table 6 proved that there is a good correlation between the results obtained using the proposed disposable stochastic sensors for the assay of PAA and SAA in whole blood and saliva samples.

Paired *t-*tests at 99.00% confidence level were performed for the assay of each interleukin, using all results shown in Table 5 and Table 6. All values calculated for the paired t-test at the 99.00% confidence level were less than the tabulated theoretical value: 4.032: for PAA the value recorded was 2.78 and for SAA, the value recorded was 2.97. Accordingly, there is no statistically significant difference between the results obtained using the proposed stochastic sensors. Accordingly, the proposed disposable stochastic sensors and the screening methods for PAA and SAA can be successful used for the analysis of biologic samples.

## 4. Conclusions

Three new disposable stochastic sensors were proposed for the determination of PAA and SAA in biologic samples. The sensors were highly reliable and sensitive. The most important benefits of the proposed method include: very low cost of fabrication, no pretreatment of the biologic sample was needed before measurements, the analysis was fast and reliable.

## Figures and Tables

**Table 1:** Response characteristics of the stochastic sensors used for determination of pancreatic α-amylase (PAA).

Microsensors Based On	Calibration Equation and Correlation Coefficient (r) *	Linear Concentration Range (U mL^−1^)	t_off_ (s)	Sensitivity (s^−1^/U mL^−1^)	Limit of Determination (U mL^−1^)
α-CD/Ag	1/t_on_ = 0.02 + 2.49(± 0.05) × 10^3^ × C r = 0.9993	1.00 × 10^−7^–1.00 × 10^3^	0.6 ± 0.1	2.49 × 10^3^	1.00 × 10^−7^
α-CD/AgC	1/t_on_ =0.03 + 1.85(± 0.07) × 10^3^ × C r = 0.9999	1.00 × 10^−7^–1.00 × 10^3^	1.2 ± 0.2	1.85 × 10^3^	1.00 × 10^−7^
α-CD/AgC/Ag	1/t_on_ =0.03 + 1.72(± 0.02) × 10^3^ × C r = 0.9999	1.00 × 10^−7^–1.00 × 10^3^	1.2 ± 0.1	1.72 × 10^3^	1.00 × 10^−7^

* <C> = U L^−1^; <t_on_> = s.

**Table 2:** Response characteristics of the stochastic sensors used for determination of salivary α-amylase (SAA).

Microsensors Based On	Calibration Equation and Correlation Coefficient (r) *	Linear Concentration Range (U mL^−1^)	t_off_ (s)	Sensitivity (s^−1^/U mL^−1^)	Limit of Determination (U mL^−1^)
α-CD/Ag	1/t_on_ = 0.01 + 7.07(± 0.05) × 10^8^ × C r = 0.9994	1.5 × 10^−9^–1.5 × 10^2^	0.6 ± 0.1	7.07 × 10^8^	1.5 × 10^−9^
α-CD/AgC	1/t_on_ =0.01 + 3.10(± 0.05) × 10^6^ × C r = 0.9013	1.5 × 10^−15^–1.5 × 10^2^	1.0 ± 0.2	3.10 × 10^6^	1.5 × 10^−15^
α-CD/AgC/Ag	1/t_on_ = 0.01 + 1.20(± 0.05) × 10^7^ × C r = 0.9704	1.5 × 10^−15^–1.5 × 10^2^	0.5 ± 0.1	1.20 × 10^7^	1.5 × 10^−15^

* <C> = U mL^−1^; <t_on_> = s.

**Table 3:** Recovery of pancreatic α-amylase (PAA) in whole blood samples (N = 10).

Microsensors Based On	%, Recovery of PAA
α-CD/Ag	99.78 ± 0.03
α-CD/AgC	99.73 ± 0.05
α-CD/AgC/Ag	99.85 ± 0.02

**Table 4:** Recovery of salivary α-amylase (SAA) in saliva samples (N = 10).

Microsensors Based On	%, Recovery of SAA
α-CD/Ag	99.85 ± 0.05
α-CD/AgC	99.80 ± 0.03
α-CD/AgC/Ag	99.98 ± 0.02

**Table 5:** Determination of pancreatic α-amylase (PAA) in whole blood samples (N = 10).

Microsensors Based On	PAA, U L^−1^				
	Sample 1	Sample 2	Sample 3	Sample 4	Sample 5
α-CD/Ag	43.77 ± 0.05	23.13 ± 0.04	27.35 ± 0.02	42.80 ± 0.04	80.01 ± 0.05
α-CD/AgC	43.14 ± 0.07	24.21 ± 0.04	28.49 ± 0.02	43.77 ± 0.05	80.52 ± 0.03
α-CD/AgC/Ag	42.14 ± 0.03	23.87 ± 0.03	28.53 ± 0.04	42.61 ± 0.02	80.42 ± 0.02

**Table 6:** Determination of salivary α-amylase (SAA) in saliva samples (N = 10).

Microsensors Based On	SAA, U mL^−1^				
	Sample 1	Sample 2	Sample 3	Sample 4	Sample 5
α-CD/Ag	86.78 ± 0.03	32.70 ± 0.03	59.90 ± 0.05	79.88 ± 0.03	53.77 ± 0.03
α-CD/AgC	86.30 ± 0.04	32.88 ± 0.05	59.99 ± 0.05	79.23 ± 0.03	53.58 ± 0.04
α-CD/AgC/Ag	86.90 ± 0.05	32.87 ± 0.01	59.55 ± 0.02	79.33 ± 0.04	54.05 ± 0.01
